# Ignition Delay Time Measurements of Substituted Phenol Additives in a Toluene Reference Fuel

**DOI:** 10.1021/acsomega.4c06985

**Published:** 2024-11-01

**Authors:** Grace Trombley, Elisa Toulson

**Affiliations:** Alternative Fuels and Combustion Laboratory, Michigan State University, 1497 Engineering Research Ct, East Lansing, Michigan 48824-6206, United States

## Abstract

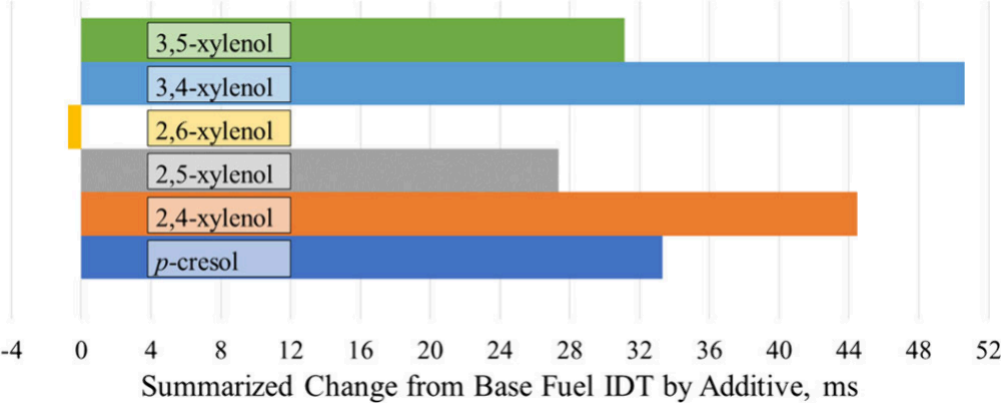

Using a rapid compression machine, fuel autoignition resistance can be quantified by the ignition delay time measurements of homogeneous mixtures. As the occurrence and intensity of knock in spark ignition engines are related to autoignition resistance, ignition delay time measurements give valuable insight into fundamental fuel combustion properties that can be used to predict undesirable combustion behavior. Therefore, the work presented in this paper aims to understand the autoignition resistance of a gasoline surrogate fuel and how it is affected by the addition of substituted phenol additives from ignition delay time measurements in a rapid compression machine. Six substituted phenols were tested: *p*-cresol, 2,4-xylenol, 2,5-xylenol, 2,6-xylenol, 3,4-xylenol, and 3,5-xylenol. Lean and stoichiometric mixtures, as well as stoichiometric mixtures with N_2_ dilution, were studied at engine-relevant conditions of 20 bar between 700 and 950 K. It was found that most additives were able to lengthen the base fuel ignition delay time at high and low temperatures, but that the most effective had two methyl groups located adjacent to each other.

## Introduction

Combined with engine downsizing and turbocharging, an increase in fuel research octane number (RON) can be used in light-duty vehicles to meet emissions standards set by the United States Environmental Protection Agency (EPA).^1^ Furthermore, efforts to combine turbocharging with hybrid vehicle technology has shown that an increase in RON would allow an increase in fuel efficiency.^2^ Since gasoline-fueled vehicles comprise much of the light-duty vehicle fleet in the US and will continue to occupy a nontrivial portion of the global transport sector for years to come,^3−5^ a fuel that is suitable as both a drop-in for existing vehicles and high-efficiency engines would have economic and environmental benefits.^6^

Oxygenated components have been added to commercial-grade gasoline to meet engine octane number (ON) requirements, such as methyl *tert*-butyl ether (MTBE), ethanol, and aromatic hydrocarbons.^5,7−9^ In the United States, MTBE has been phased out of use with ethanol often being used in favor at relatively high concentrations (up to 10% by volume in midgrade pump gasoline).^10,11^ Aromatics, such as toluene have also been used to boost fuel ON and sensitivity (*S*), which is the difference between the measured RON and motor octane number (MON), eq 1.^12^ Sensitivity has been found to be related to autoignition resistance, where the addition of a more sensitive fuel to gasoline surrogates has been found to increase autoignition resistance with an increase in pressure.^13^ As ethanol and toluene have RONs of 109 and 120, respectively, similar additives may also be able to increase gasoline ON when added at even smaller volumes.^14^
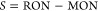
1While RON and MON are commonly used metrics to grade gasoline, their testing procedures are relatively costly and do not represent fuel performance over an entire engine operating range.^15,16^ Ignition delay time (IDT) measurements can be used to understand a fuel’s autoignition resistance over the same conditions, as well as at the high temperatures and pressures experienced in the end-gas region of the combustion chamber.^16,17^ By characterizing ignition resistance over an engine-relevant temperature range, the potential for these additives to be octane-enhancing can be estimated. Therefore, this paper aims to understand the autoignition resistance of a Toluene Reference Fuel (TRF) with substituted phenol additives using IDT measurements from a Rapid Compression Machine (RCM).

Modeling done by Zhang et al.^18^ found that 2,4-xylenol was able to lengthen the IDT of stoichiometric *n*-butane/air mixtures when blended with *n*-butane at 2% for temperatures higher than the negative temperature coefficient (NTC) region. In the same study, *p*-cresol was also able to lengthen the IDT over a smaller range of temperatures above the NTC region. For both additives, hydrogen abstraction reactions initiated consumption and were followed by disproportionation reactions that resulted in a relatively stable conjugated ketone. Since abstraction reactions began at the methyl groups on each additive, the authors found that the presence of two methyl groups on 2,4-xylenol was responsible for its stronger antiknock effect than *p*-cresol. This is supported by kinetic modeling of C7–C11 methylated aromatics by Kukkadapu et al.,^19^ who validated their mechanism against IDT measurements. A review of aromatic combustion chemistry by Jin et al.^20^ also detailed the importance of methyl group location on the aromatic ring, due to their ability to interact with each other without necessarily influencing the ring structure, especially at low-temperature. Modeling of toluene reference fuels by Andrae^21^ found strong temperature dependence of these interactions, which affects *S* and IDT. Polikarpov et al.^22^ suggested that intermolecular interactions observed between prenol’s hydroxyl group and an olefin^23^ can also be found in other chemical structures, and found that, in the 5- and 6-membered rings studied, methyl substitution decreased *S*.^22^

While longer IDTs resulting from an increase in fuel aromatic content are useful, this can also increase soot formation.^24^ Guerrero Peña et al.^24^ found that increasing the number of methyl groups on an aromatic ring corresponds to increased soot formation. This trade-off between IDT and soot formation has been studied and found to be a function of the aromatic itself and its interactions with the remaining fuel components.^12^ When added at 10% by volume to a gasoline surrogate, 2,4-xylenol was able to increase blending RON and MON and produced lower particulate matter (PM) and particulate number (PN) emissions than predicted by the particulate matter index (PMI),^25^ shown in eq 2,^26^ where *i* is the fuel component, DBE is the double bond equivalent calculated by eq 3, *P*_vap_ is the vapor pressure estimated by the Clausius–Clapeyron equation at 443 K, and *w*_*i*_ is the component mass fraction. Equation 3 is calculated for each hydrocarbon component of the form C_*x*_H_*y*_O_*z*_.
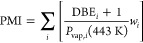
2
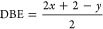
3

Table 1 shows the PMI calculated for all fuels included in this study with each of the substituted phenols increasing the value significantly. PMI = 1.5 ± 0.69 bar^–1^ has been reported for gasoline, where lower PMIs would be favored for emissions reduction.^27^ As the substituted phenols in this work did increase the PMI of the base fuel significantly, they were added at low levels (2% by mole) to the base fuel to reduce their soot-forming impact while capturing their ON-enhancing and IDT lengthening properties.^25,28^

**Table 1:** Fuel Compositions

	fuel constituent, by % mole									
surrogate	isooctane	toluene	*n*-heptane	*p*-cresol	2,4-xylenol	2,5-xylenol	2,6-xylenol	3,4-xylenol	3,5-xylenol	PMI, bar^–1^
Base Fuel	55.65	27.44	16.91							0.44
Fuel p	54.53	26.89	16.58	2						0.75
Fuel 2,4					2					0.82
Fuel 2,5						2				0.82
Fuel 2,6							2			0.75
Fuel 3,4								2		0.99
Fuel 3,5									2	0.91

## Experimental Methods

### Fuel Composition

**Figure 1:**
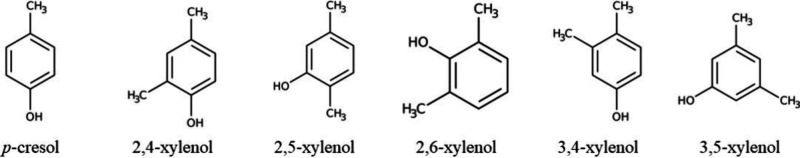
Six substituted phenols tested.

The base fuel used in these experiments was a TRF, consisting of 63% isooctane, 20% toluene, and 17% *n*-heptane by volume, with IDT behavior representative of a midgrade 87 octane gasoline.^29^ To the base fuel, six different substituted phenols were added at 2% by mole to create a total of seven mixtures. The structures of the tested additives: *p*-cresol, 2,4-xylenol, 2,5-xylenol, 2,6-xylenol, 3,4-xylenol, and 3,5-xylenol, are shown in Figure 1, and each mixture’s composition is detailed in Table 1. IDTs of *p*-cresol and 2,6-xylenol were first published in ref (30) and 2,4-xylenol and 3,5-xylenol were published in ref (31). The findings of these papers are included in the following discussion of additive chemical structure on IDT, along with new measurements of 2,5-xylenol and 3,4-xylenol that are presented in the Results and Discussion. 2,3-Xylenol was also considered as a potential additive, but measurements were not performed as 2,3-xylenol did not dissolve completely in the TRF when added at 2% by mole at room temperature, so a homogeneous fuel mixture was not possible in the system detailed in the following sections.

### Rapid Compression Machine

**Figure 2:**
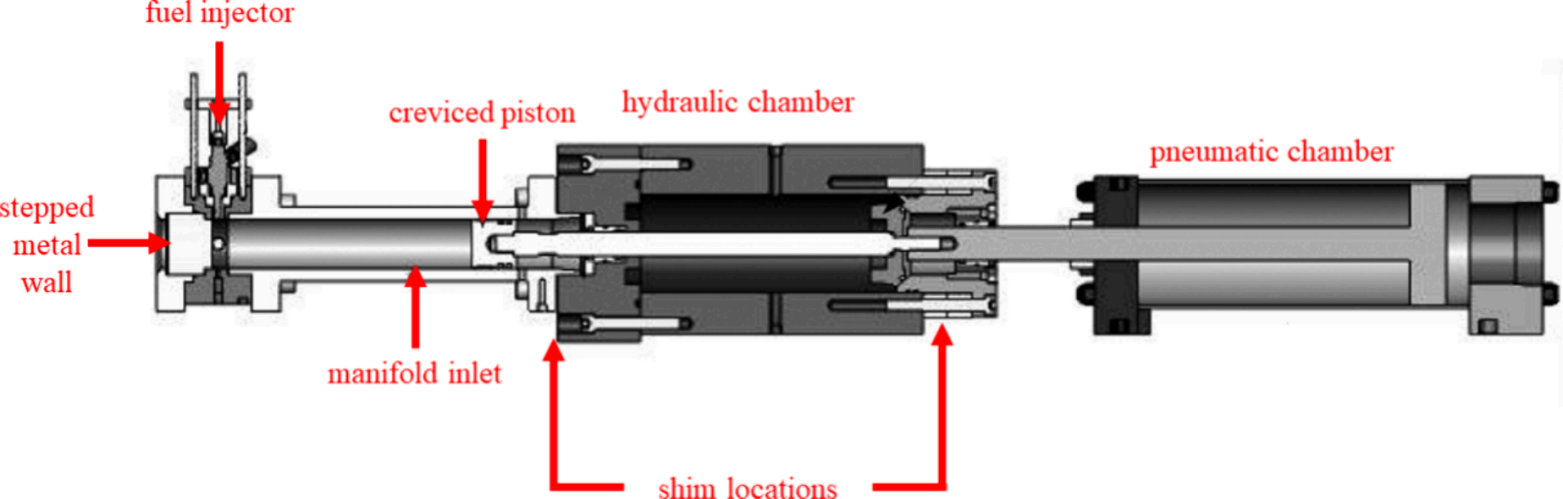
Schematic of the rapid compression machine with important features labeled.

The RCM used in this study is housed at Michigan State University, and its ability to measure IDTs of a wide range of fuels down to 3 ms and up to 100 ms has been documented.^31−38^ This RCM has a compression time of ∼30 ms, depending on the compression ratio (CR), and its features are labeled in Figure 2. A pressurized pneumatic chamber drives the piston during the compression stroke, and a pressurized hydraulic chamber stops the piston. CRs of 6–17 are achieved by flipping the stepped metal wall at the end of the combustion chamber and adjusting the number of shims ahead of and behind the hydraulic chamber to change the volumes at bottom dead center (BDC) and top dead center (TDC). The reactants are mixed inside the combustion chamber using the Direct Test Chamber (DTC) approach validated by Allen et al.^32^ and Chinnathambi,^39^ where gaseous air and diluents are added to the heated combustion chamber (>120 °C) through the manifold and a calibrated fuel injector is used to add liquid fuel to the combustion chamber. Using the law of partial pressures, the reactants could be assumed homogeneous when the fuel partial pressure was lower than its vapor pressure and was given sufficient time to evaporate and mix with gaseous reactants (2 min) ahead of the compression stroke.^39^ The creviced piston served to reduce roll-up vortexes during compression and prevent heat transfer between the homogeneous mixture and combustion chamber wall, such that an “adiabatic core” could be assumed for 100 ms after the piston reached TDC.^40−42^

### Ignition Delay Time

IDT was defined as the difference in time between the arrival of the piston at the TDC and the maximum rate of pressure rise due to autoignition. Using measurements from a Kistler 6125C piezoelectric pressure transducer installed in the combustion chamber, TDC and ignition were identified from the derivatives of the pressure trace with respect to time. TDC was the first instance of the pressure rise rate equal to zero (d*P*/d*t* = 0) after compression began, and the instant of the maximum pressure rise rate was defined as ignition. The temperature at TDC was calculated using the adiabatic core hypothesis, eq 4, which assumes minimal mixing between the relatively cool combustion chamber walls and the adiabatic “core” region in the center of the combustion chamber.^41,43^
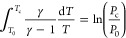
4The known initial temperature (*T*_0_), ratio of specific heats (γ), compressed pressure (*P*_c_), and initial pressure (*P*_0_) allow the compressed temperature (*T*_c_) to be found. This relation has been found to hold true up to 100 ms after the piston reaches TDC, where roll-up vortexes generated during the compression stroke are still contained by the creviced piston.^41,42^ Heat losses during the compression stroke are accounted for in eq 4 by *P*_c_, where fuel composition is accounted for by γ, and postcompression heat losses are negligible for 100 ms after TDC because of the creviced piston used.^38,41^

Importantly, each reactive test performed was compared against a nonreactive test at the same initial conditions to ensure that the measurements presented here were free from significant combustion reactions during the compression stroke, which is shown by no deviation from the nonreactive pressure trace on behalf of the reactive pressure trace.^44^ Nonreactive pressure traces were performed at every CR, φ, and the dilution ratio by replacing the O_2_ portion of each mixture with N_2_. Sung and Curran^44^ highlight that matching reactive and nonreactive pressure traces do not necessarily exclude radical initiation reactions during the compression stroke that may affect combustion chemistry after the piston reaches TDC, which can be better understood with chemical kinetic modeling during the compression stroke. By this justification, the RCM here has been found to measure IDT down to 3 ms without reactive chemistry affecting the pressure trace before the piston’s arrival at TDC.^30,31,33,34,45^

### Test Conditions

For all experiments, a dry air mixture of 79% N_2_ and 21% O_2_ was used. To simulate exhaust gas recirculation (EGR), N_2_ was used to eliminate chemistry effects but include the cooling effect of real EGR products.^30,31^ Since N_2_ is an inert primary constituent of real combustion residuals of stoichiometric mixtures, its usage as “EGR” in this case is justified and serves to mimic its real and passive presence in practical combustors. Furthermore, using an inert gas, like N_2_, to simulate EGR prevents changes in combustion chemistry due to the oxidizer and diluents, and instead the differences in measured combustion parameters can be attributed to the fuels studied. As the goal of this study was to characterize differences in combustion performance of a TRF with substituted phenol additives with variation in pressure (*P*), temperature (*T*), and φ, eliminating variations in combustion chemistry due to air and diluent composition allows more insight into the aromatic chemistry responsible for differences in combustion properties. IDT measurements occurred at 20 bar and 700–950 K, which are included in the conditions detailed in Table 2.

**Table 2:** IDT Test Conditions^a^

Pressure, bar	Temperature, K	φ	N_2_ dilution % at φ = 1
20	700	0.6	0
	750*		
	800*	0.8	15
	850		
	900	1.0	30
	950*		

a An ‘*’ next to any compressed temperature indicates that not all measurements performed were free from precompression reactions, so only the measurements free from precompression reactions are presented.

## Results and Discussion

### IDT Results

**Figure 3:**
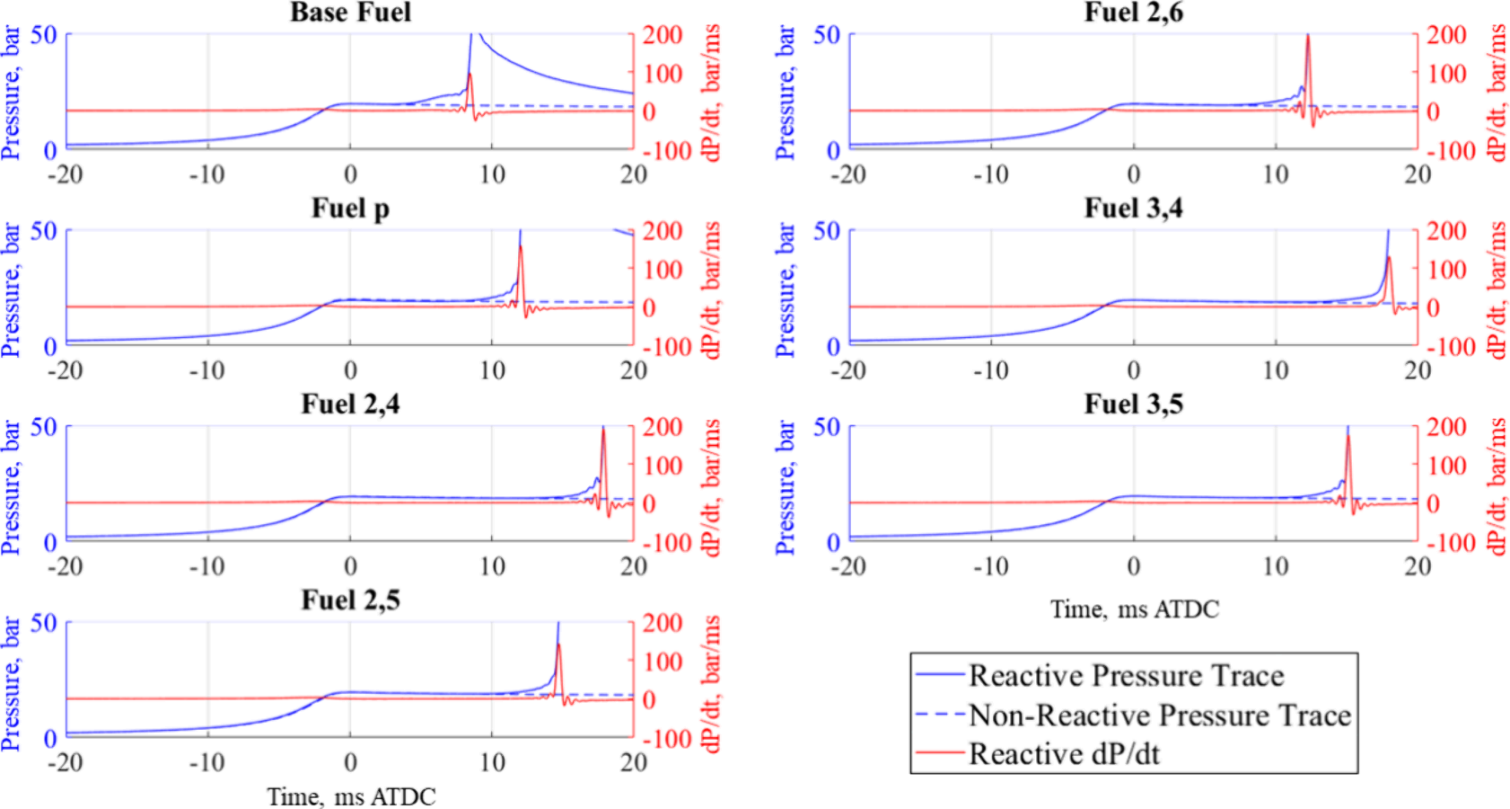
Reactive and nonreactive pressure traces for each mixture, as well as d*P*/d*t* corresponding to the reactive pressure trace when φ = 0.8 @ 700 K and 20 bar.

**Figure 4:**
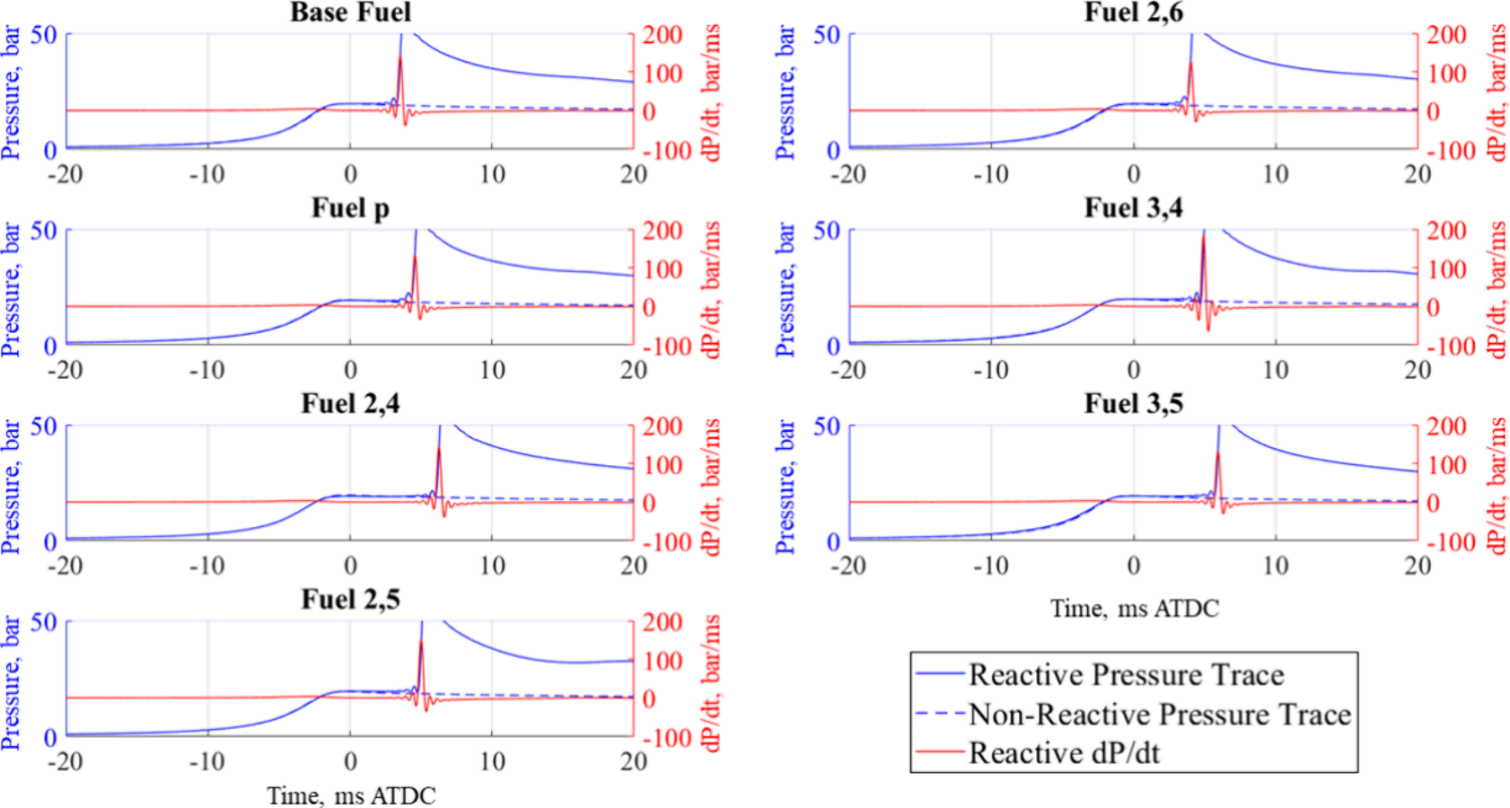
Reactive and nonreactive pressure traces, as well as d*P*/d*t* corresponding to the reactive pressure trace when φ = 0.8 @ 900 K and 20 bar.

IDT measurements are presented and discussed according to additive, φ, and dilution in the following subsections. In all graphs, solid lines are used to connect measurements at 50 K intervals and dotted lines are used to connect measurements at 100 K intervals, which occurred when measurements could not be taken at the 50 K interval. At either 750 or 800 K, measurements could not be taken for each fuel because of significant reactions occurring ahead of the piston reaching TDC. These points exist within the region spanned by the dotted line in each graph, which means that the actual IDT would likely be much faster than the IDT trend shown by the dotted lines. A distinct NTC region would be present between 700 and 850 K, depending on the additive used, if this prediction were to hold true. For some additives, φ, and dilution levels, the IDT at 950 K was not long enough to exclude precompression reactions, so the only measurements presented were sufficiently long to exclude significant combustion reactions ahead of the piston reaching TDC. Each of the IDT measurements presented in the upcoming sections and in refs (30) and (31) were taken at constant volume, where this was verified by reactive and nonreactive pressure traces. Examples of this comparison are shown in Figure 3 and Figure 4 for φ = 0.8 at 20 bar and 700 and 900 K, respectively.

**Figure 5:**
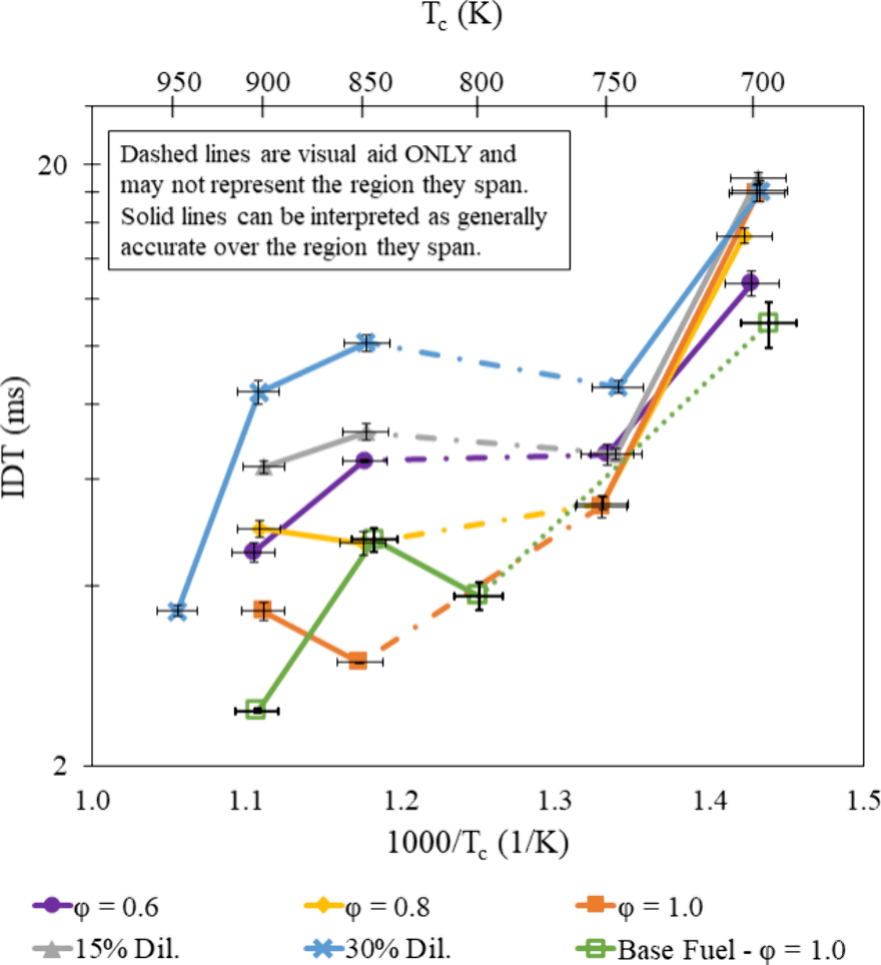
IDT measurements of Fuel 2,5 at 20 bar with the stoichiometric base fuel results.

At all test conditions, at least three reactive pressure traces were taken, and the average of the IDT measurements are presented in Figure 5–Figure 11. On each graph, error bars equal to the standard deviation of these measurements are shown. All IDT measurements were within 10% of the mean. Sources of uncertainty in the final temperature are the initial mixture temperature (±5 K), initial pressure (±0.08%), compressed pressure (±0.3 bar), and fuel mass (±1.5%). These measurement uncertainties result in an uncertainty of ±1.25% in the compressed temperature calculated by eq 4 and are represented as horizontal error bars in Figure 5–Figure 11.

### Additive Effects

**Figure 6:**
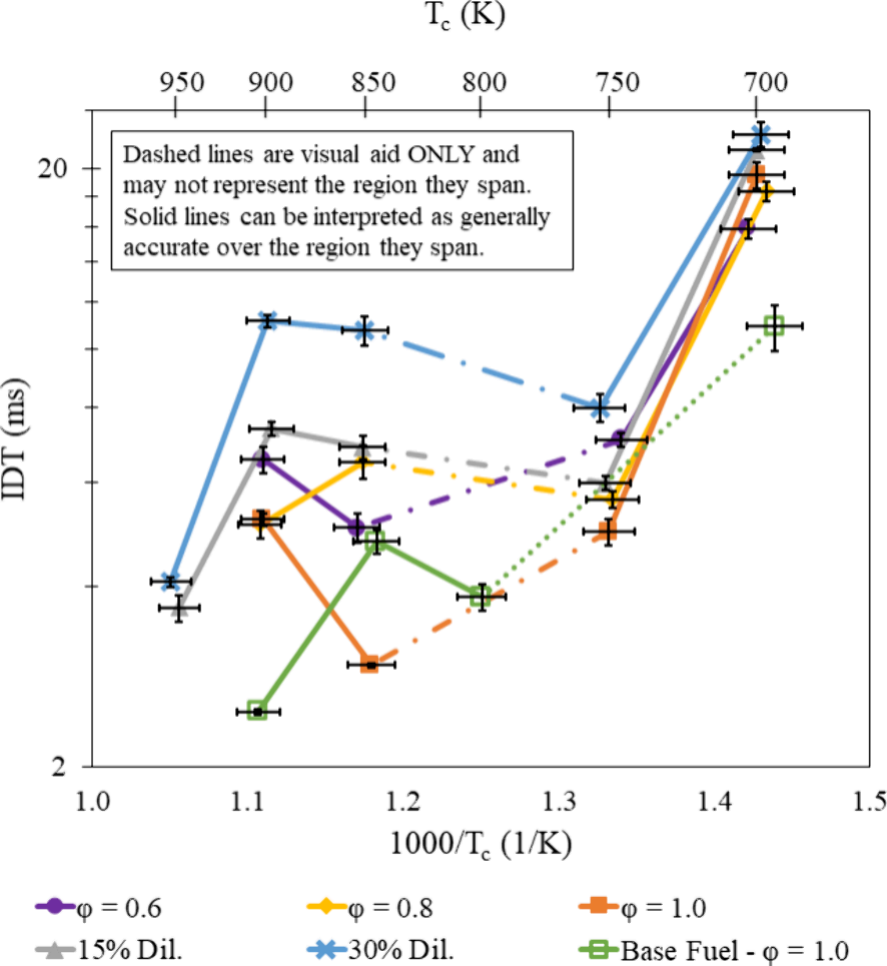
IDT measurements of Fuel 3,4 at 20 bar with the stoichiometric base fuel results.

IDT measurements of Fuel 2,5 and Fuel 3,4 with respect to temperature as φ and dilution were varied are shown in Figure 5 and Figure 6, respectively. At low temperatures, IDTs of all φ and dilution ratios are relatively similar for both fuels, and an increase in temperature corresponds to a larger spread of IDTs. The sharp decrease in IDT as the temperature increases from 700 to 750 K agrees with the measurements of Kukkadapu et al.,^46^ who studied the same TRF at 20 bar. Furthermore, neither Fuel 2,5 nor Fuel 3,4 were able to resist precompression reactions at 800 K and this agrees with the data from Kukkadapu et al.,^46^ which found the NTC region to occur between 750 and 850 K. Looking at the TRF results from the RCM used in the current study,^30^ IDT measurements at 800 K were possible but measurements at 750 K were not.

The *n*-heptane component of the base fuel contributes to two-stage ignition phenomena at low-temperatures, as shown in Figure 3. A series of hydrogen abstraction reactions from *n*-heptane and O_2_ additions leads to the production of a ketohydroperoxide and OH radical.^47^ In this branching sequence, decomposition of ketohydroperoxide is relatively slow, results in another OH radical, and determines the rate of branching. Once *n*-heptane is consumed, the OH concentration increases rapidly and is followed by consumption of intermediate species by exothermic reactions, corresponding to ignition.^48^ As the temperature is increased, thermal decomposition drives ignition to occur much faster than the hydrogen abstraction reactions can facilitate, which is why two-stage ignition is not present for the base fuel at 900 K in Figure 4. The additive-containing mixtures in Figure 3 do not demonstrate prominent two-stage ignition behavior because hydrogen abstraction reactions by each substituted phenol serve to form a conjugated ketone. As the conjugated ketones formed are relatively stable at low temperatures, they serve to resist low- and intermediate-temperature reactions that would result in the pressure rise ahead of autoignition as seen in the base fuel. When the reactant temperature is increased, β-scission takes over through the narrow temperature range of the NTC region where HO_2_ radical decomposition exceeds production, causing the IDT to increase with temperature. By replacement of *n*-heptane within the base fuel with substituted phenols, the aromatic content of each additive-containing mixture serves to resist low-temperature reactions and delay ignition until sufficiently high temperatures are achieved for β-scission to occur. As chemical kinetic analysis was not used in the current work, differences in overall IDT with respect to each substituted phenol’s methyl group location cannot be determined. However, it can be expected that the shift in the NTC region of the base fuel to a higher temperature within the range 750–850 K caused by the addition of 2,4-xylenol, 2,5-xylenol, 3,4-xylenol, or 3,5-xylenol is a result of the additives’ ability to consume OH radicals from the *n*-heptane.^13^

### Equivalence Ratio Effects

**Figure 7:**
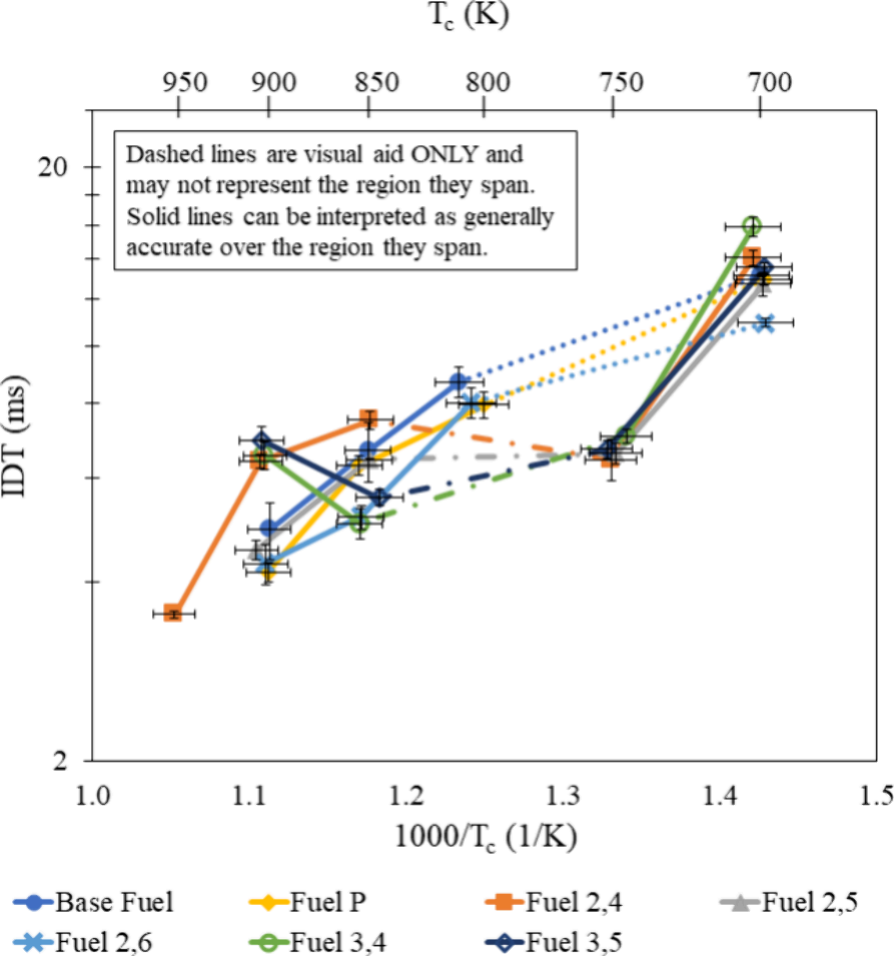
IDT measurements of all fuel mixtures tested at φ = 0.6 and 20 bar, with results from refs (30) and (31).

As φ increased from 0.6 to 1.0, the lengthening effect of all additives at 700 K increased such that the base fuel had the shortest IDT at this condition, shown in Figure 7–Figure 9. This same trend also holds true at high temperatures but does not apply to the NTC temperature range. At 800 K, the base fuel had some of the longest IDTs measured, which can be attributed to aromatic chemistry associated with the additives’ chemical structures and is discussed in the Aromatic Chemistry section. As the TRF and *p*-cresol- and 2,6-xylenol-containing mixtures were the only fuels that had measurable IDTs at 800 K and not 750 K, one can expect that *p*-cresol and 2,6-xylenol did not shift the NTC region of the TRF while the other four additives did. For these three mixtures, the IDT decreases as the temperature increased from 850 to 900 K, whereas the remaining additive mixtures (2,4-xylenol, 2,5-xylenol, 2,4-xylenol, and 3,5-xylenol) generally see an increase in IDT from 850 to 900 K, except for the 3,4-xylenol mixture at φ = 0.8, indicating a shift in the NTC region caused by each additive.

**Figure 8:**
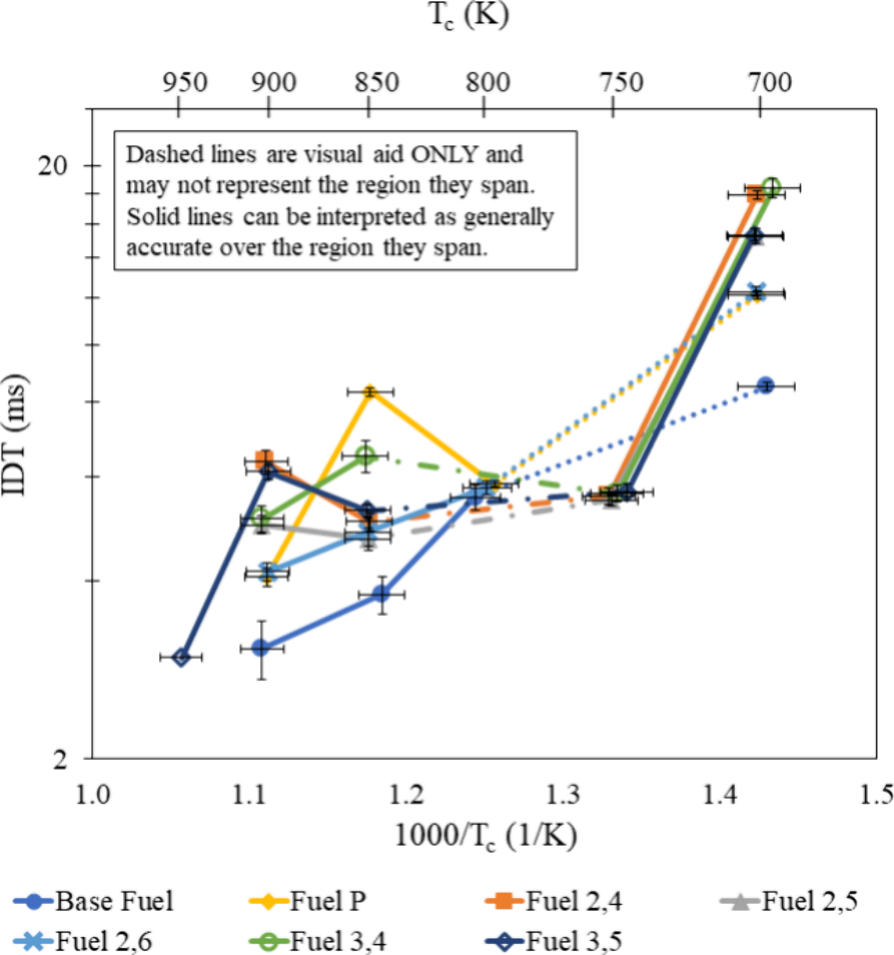
IDT measurements of all fuel mixtures tested at φ = 0.8 and 20 bar, with results from refs (30) and (31).

**Figure 9:**
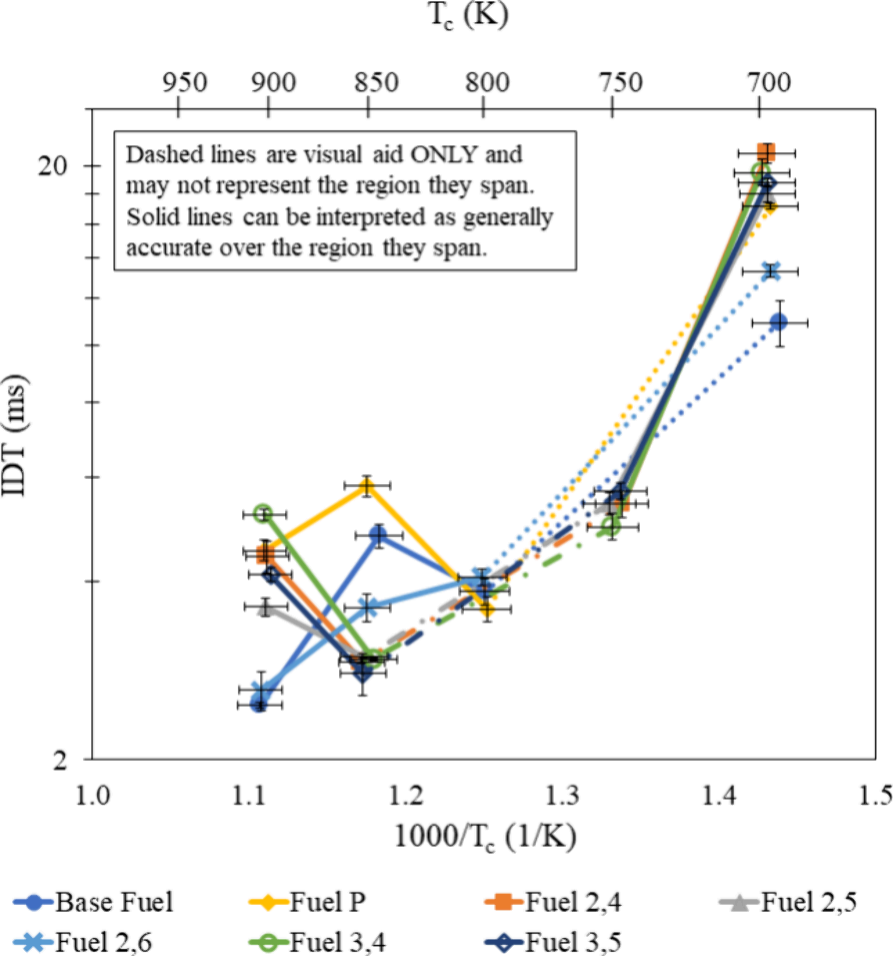
IDT measurements of all fuel mixtures tested at φ = 1.0 and 20 bar, with results from refs (30) and (31).

### Dilution Effects

**Figure 10:**
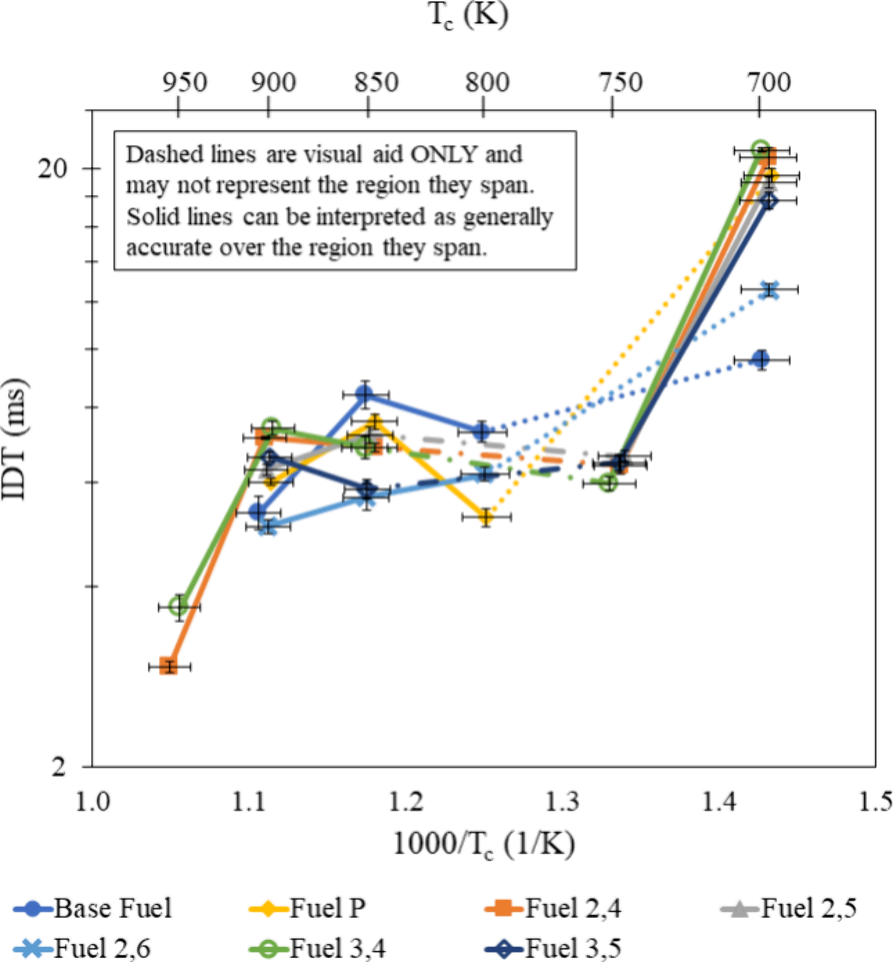
IDT measurements of all fuel mixtures tested at φ = 1.0, 15% dilution and 20 bar, with results from refs (30) and (31).

**Figure 11:**
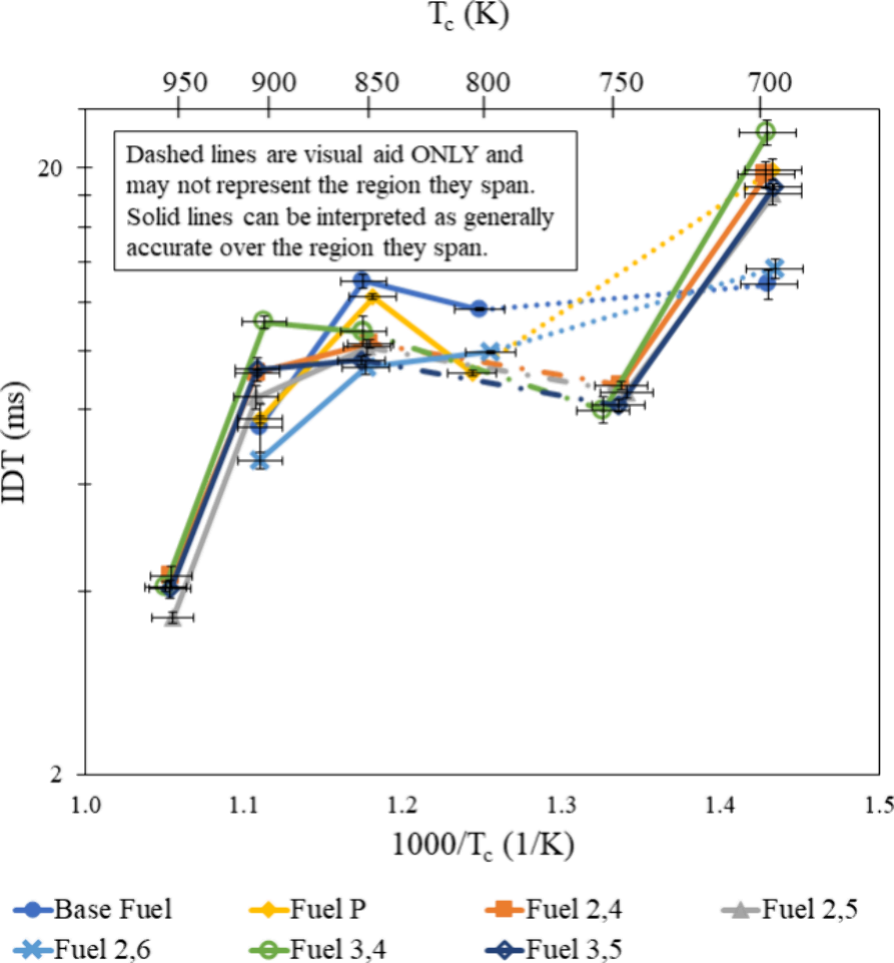
IDT measurements of all fuel mixtures tested at φ = 1.0, 30% dilution and 20 bar, with results from refs (30) and (31).

Figure 5 and Figure 6 show that an increase in the N_2_ dilution resulted in longer IDTs, especially at high temperatures. When added to stoichiometric reactants, N_2_ serves to lower the combustion temperature while remaining inert to simulate the cooling effect of EGR. By adding N_2_ in place of actual EGR products, the results in Figure 10 and Figure 11 serve to show that lowering the combustion temperature of each additive-containing mixture will result in different IDT behavior because of each additive’s unique methyl and hydroxyl group locations. Compared to the stoichiometric air/fuel mixture, the N_2_-diluted stoichiometric mixtures have flatter NTC regions and do not appear to have shifted NTC regions from their respective stoichiometric nondiluted mixture.

### Aromatic Chemistry

Variation of base fuel IDT with different substituted phenol additives illustrates the significance of aromatic structure on autoignition of a gasoline surrogate under engine-relevant conditions, even when included at small rates (2% by mole). As established by literature reviewed in the Introduction, hydrogen abstraction reactions initiate consumption, and the methyl groups attached to the aromatic rings in each of the substituted phenols studied serve as a site for abstraction. *p*-Cresol only had one methyl group but had the most lengthening effect on the base fuel at midrange temperatures when φ > 0.6, near the NTC region with no N_2_ dilution. However, further increasing the temperature finds the *p*-cresol mixture to have a shorter IDT than some additives containing two methyl groups. Looking at the slopes connecting measurements for each fuel, the *p*-cresol-containing mixture does not have a uniform lengthening effect across the temperature and φ range tested, which was demonstrated by all additives and agrees with the experimental findings of Boehman et al.^49^ At low temperatures and φ > 0.6, all additives were able to significantly increase the IDT of the base fuel. When φ = 0.6, only 2,4-xylenol, 3,4-xylenol, and 3,5-xylenol were able to lengthen the base fuel IDT. The ability of all additives to increase base fuel IDT at φ = 0.8 and φ = 1.0, but only by additives with two nearby methyl groups at φ = 0.6 serves to support Zhang et al.’s^18^ claim that two methyl groups provide two locations for hydrogen abstraction reactions but indicates the importance of methyl group location relative to each other and the hydroxyl group.

**Figure 12:**
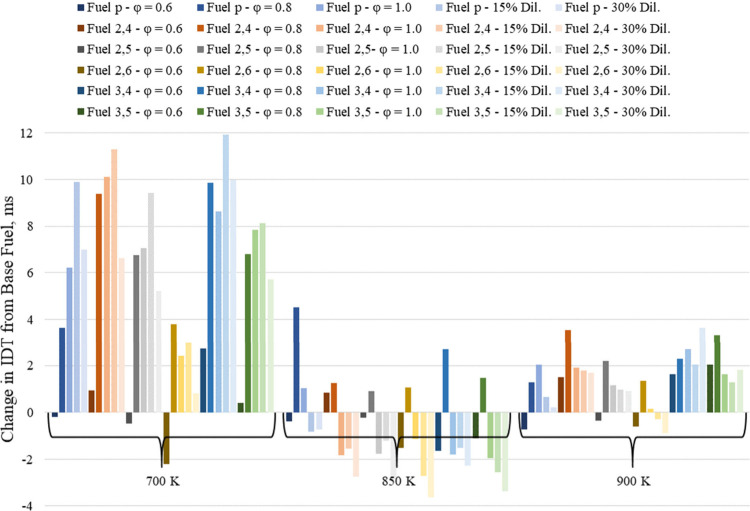
Change in IDT from the base fuel at 700, 850, and 900 K for each additive-containing fuel mixture.

Faster formation of conjugated ketones by providing two sites for hydrogen abstraction proved to have a more significant effect at higher temperatures and dilution ratios. Except for φ = 0.6 at 850 K, 2,4-xylenol and 3,5-xylenol had the most similar IDT trends at different magnitudes. These two additives have only one space between their methyl groups that is not filled by hydroxyl, but 3,5-xylenol has an additional space between its closest hydroxyl and methyl groups compared to 2,4-xylenol. 2,6-Xylenol has the most IDTs shorter than the TRF and is the only additive where the hydroxyl group is sandwiched by the methyl groups. These methyl groups are located as far apart as possible, and the additive results in more flat slopes between measurements than others who have methyl groups adjacent to the hydroxyl group (2,4-xylenol and 2,5-xylenol). Furthermore, 3,4-xylenol has two adjacent methyl groups and is able to lengthen IDT at the same conditions as 3,5-xylenol, shown in Figure 12.

**Figure 13:**
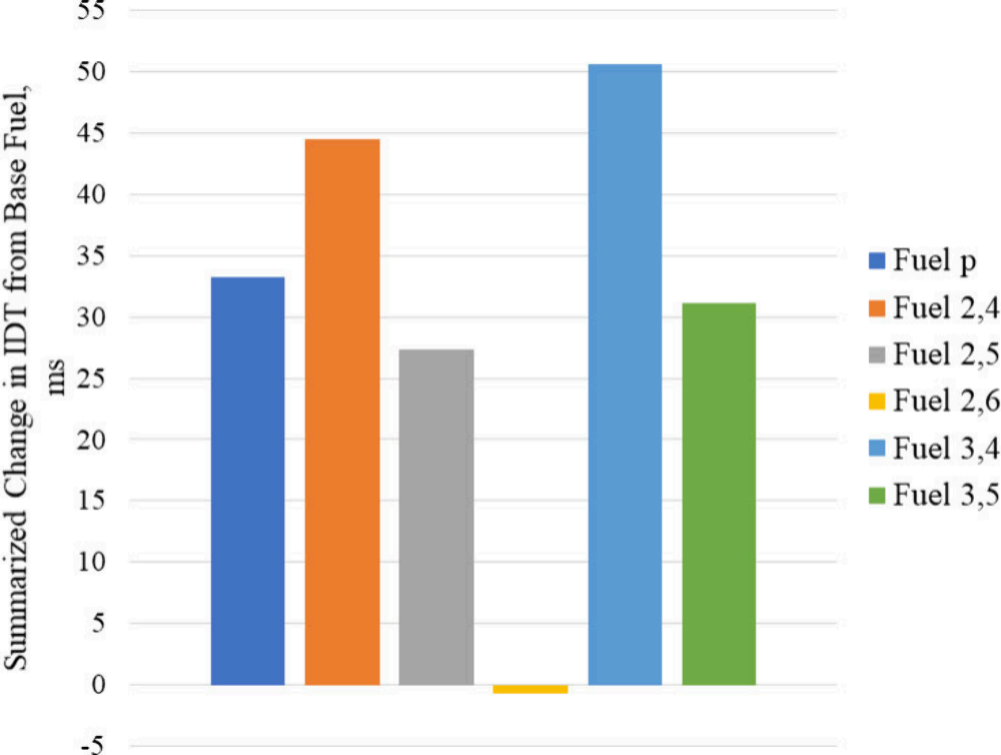
Sum of all changes in IDT from the base fuel measurements presented in Figure 12, for each fuel under all conditions.

While able to lengthen IDT under all the same conditions, except for φ = 0.6 at 850 K, the additives 2,4-xylenol, 3,4-xylenol, and 3,5-xylenol do not have the same lengthening effects. To quantify the lengthening effect of each additive across all φ values, dilution ratios, and temperatures, a summarized change in IDT from the base fuel is presented in Figure 13. The summarized value is the sum of the change in IDT from the base fuel at each φ, dilution ratio, and temperature for each fuel. These values show that 3,4-xylenol had the greatest overall lengthening effect of the additives tested and was followed by 2,4-xylenol, despite 2,4-xylenol increasing the base fuel IDT at φ = 0.6 at 850 K when 3,4-xylenol could not. Together, Figure 12 and Figure 13 suggest that additives with nearby methyl groups have the greatest lengthening effect and that the *para*-substituted methyl group on 2,4-xylenol and 3,4-xylenol contributed toward their greater autoignition resistance over the other additives tested, especially at high and low temperatures.

## Conclusions

Aromatics have long been used to increase autoignition resistance of gasoline for use in SI engines, with the desire to minimize their soot-forming behavior to meet emissions requirements. The IDT measurements presented in this paper serve to demonstrate the improved autoignition resistance of a gasoline surrogate at high and low temperatures by the addition of substituted phenols at 2% by mole. Each substituted phenol (*p*-cresol, 2,4-xylenol, 2,5-xylenol, 2,6-xylenol, 3,4-xylenol, and 3,5-xylenol) was added to its own respective mixture to help understand the importance of methyl group location on each additive’s aromatic ring as it relates to autoignition resistance. Without chemical kinetic modeling of the TRF and each additive, the reaction pathways were not used for this analysis, and IDT measurements from an RCM were used instead. IDT measurements indicate that substituted phenols at high and low temperatures are able to improve autoignition resistance but that the methyl groups must be sufficiently close to interact with each other to do so. A summation of the change in IDT from the base fuel at each φ, dilution ratio, and temperature for each additive-containing mixture found 3,4-xylenol to have the greatest increase in overall autoignition resistance from the base fuel under the conditions tested. The methyl group interaction on substituted phenols serves to generate a relatively stable conjugated ketone that helps to resist autoignition even at small volumes.

## Abbreviations

BDC: bottom dead center
CR: compression ratio
DBE: double bond equivalent
DTC: direct test chamber
EGR: exhaust gas recirculation
EPA: Environmental Protection Agency
EOC: end of compression
IDT: ignition delay time
MON: motor octane number
MTBE: methyl *tert*-butyl ether
NTC: negative temperature coefficient
ON: octane number
*P*: pressure
*P*_c_: compressed pressure
*P*_0_: initial pressure
RCM: rapid compression machine
RON: research octane number
*S*: sensitivity
*T*: temperature
*T*_c_: compressed temperature
*T*_0_: initial temperature
TDC: top dead center
TRF: toluene reference fuel
*w*: mass fraction
γ: ratio of specific heats
φ: equivalence ratio
